# Supplementary material for: Structural insights into drug transport by an aquaglyceroporin
Source: Nat Commun. 2024 May 11;15:3985. doi: 10.1038/s41467-024-48445-4 (PMC11088622; doi:10.1038/s41467-024-48445-4)
Supplement: Supplementary file 1 — Supplementary Information [file 41467_2024_48445_MOESM1_ESM.pdf]

Supplementary Information for

# Structural insights into drug transport by an aquaglyceroporin

Wanbiao Chen, Rongfeng Zou, Yi Mei, Jiawei Li, Yumi Xuan, Bing Cui, Junjie Zou, Juncheng Wang, Shaoquan Lin, Zhe Zhang, Chongyuan Wang

This file contains Supplementary Figure 1-10, Supplementary Table 1 and legends for the Supplementary Movie.

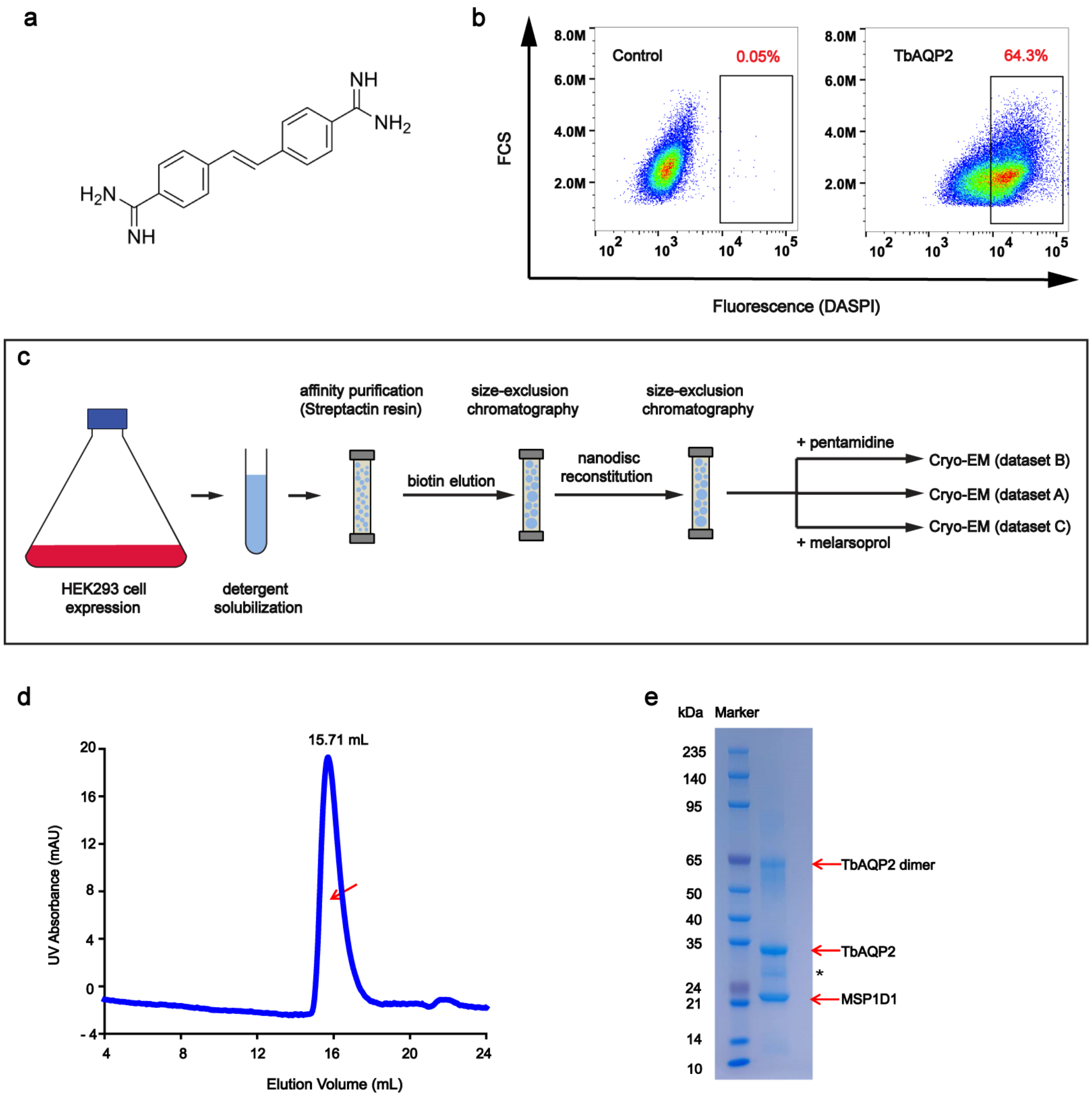


**Supplementary Fig.1 Protein expression and purification of TbAQP2.**

**a**, Structural formula of stilbamidine. **b**, Flow cytometry of HEK293S GNTI$^{-}$ cells with or without expression of TbAQP2. A representative chart from a total of three experiments is presented, and fluorescence was assessed at an excitation wavelength of 405 nm and an emission wavelength of 445±45 nm. **c**, Purification scheme. **d** and **e**, Representative SEC profile and SDS-PAGE analysis of the sample in lipid nanodisc.

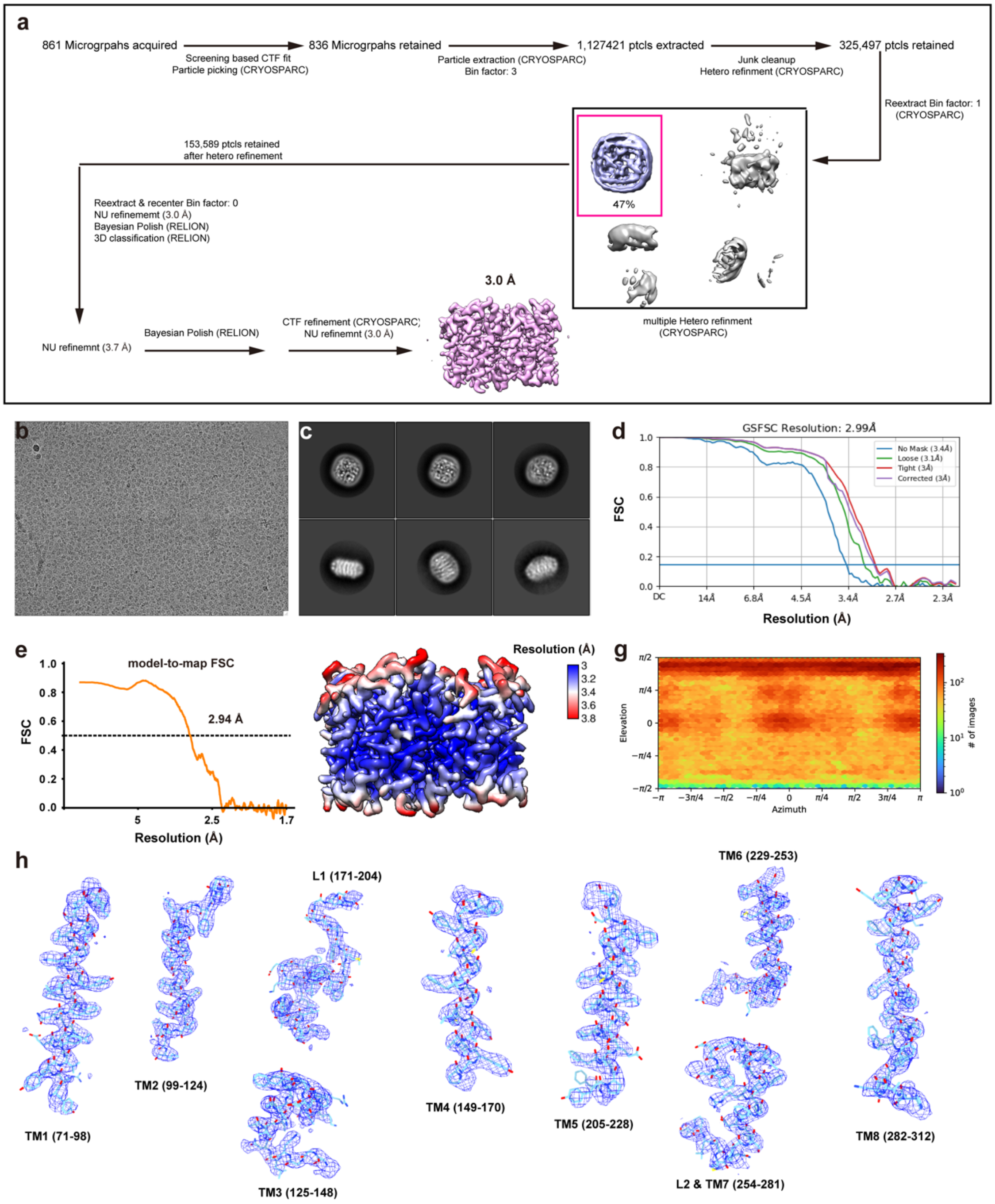


**Supplementary Fig. 2 Cryo-EM data processing and reconstruction of TbAQP2.**

**a**, Flow charts outlining the cryo-EM processing workflows. **b** and **c**, Representative cryo-EM micrographs and 2D class averages. **d-g**, Half-map FSC curves, map-to-model FSC curves, Estimations of local resolution, and the angular distributions of particles used in the final reconstructions are shown. Details can be found in Methods. **h**, Cryo-EM density. Densities (mesh rendering, 8 s contour) for indicated regions are shown in the context of the atomic model (sticks).

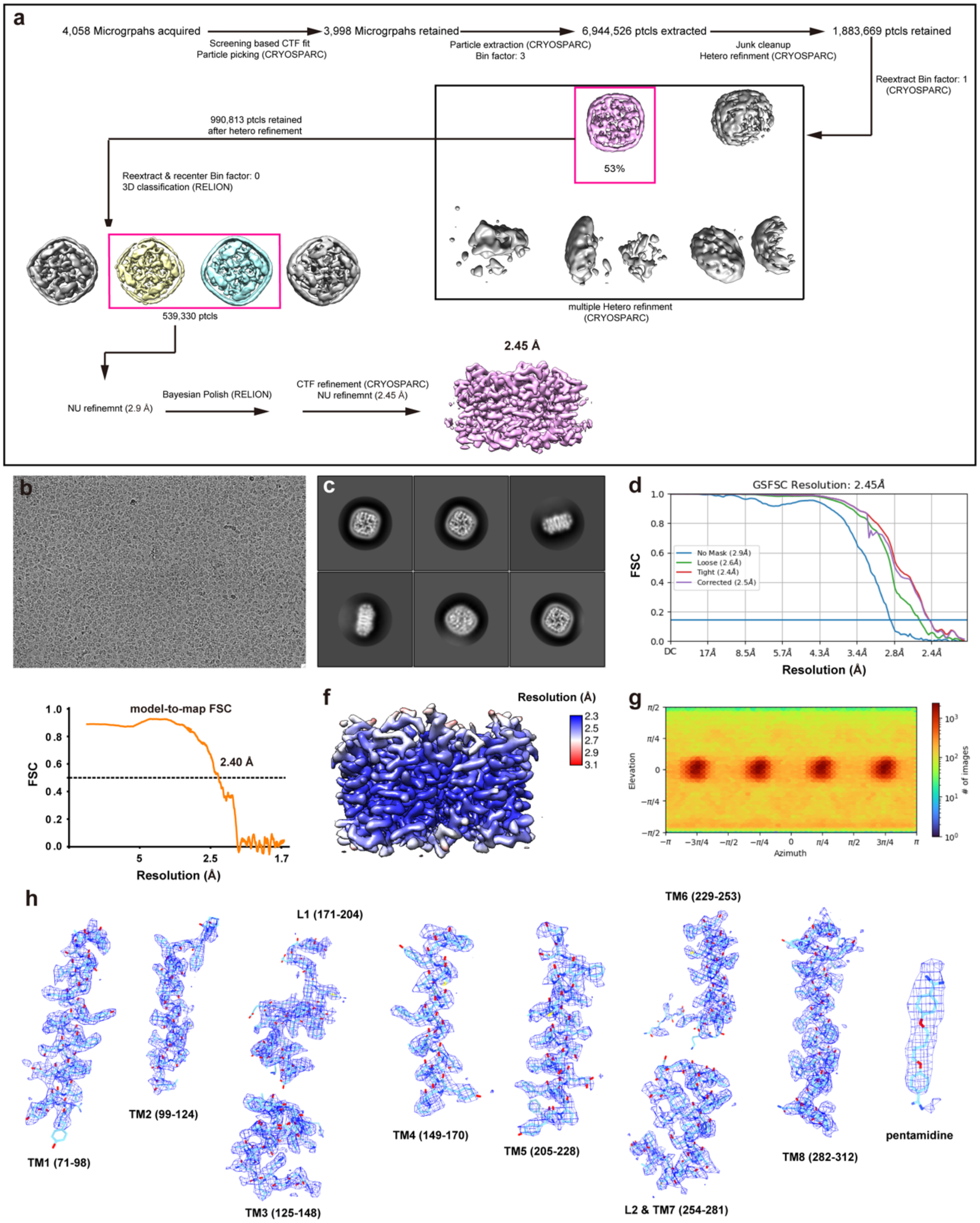


**Supplementary Fig. 3 Cryo-EM data processing and reconstruction of TbAQP2-pentamidine complex.** **a**, Flow charts outlining the cryo-EM processing workflows. **b** and **c**, Representative cryo-EM micrographs and 2D class averages. **d-g**, Half-map FSC curves, map-to-model FSC curves, Estimations of local resolution, and the angular distributions of particles used in the final reconstructions are shown. Details can be found in Methods. **h**, Cryo-EM density. Densities (mesh rendering, 8 s contour) for indicated regions are shown in the context of the atomic model (sticks).

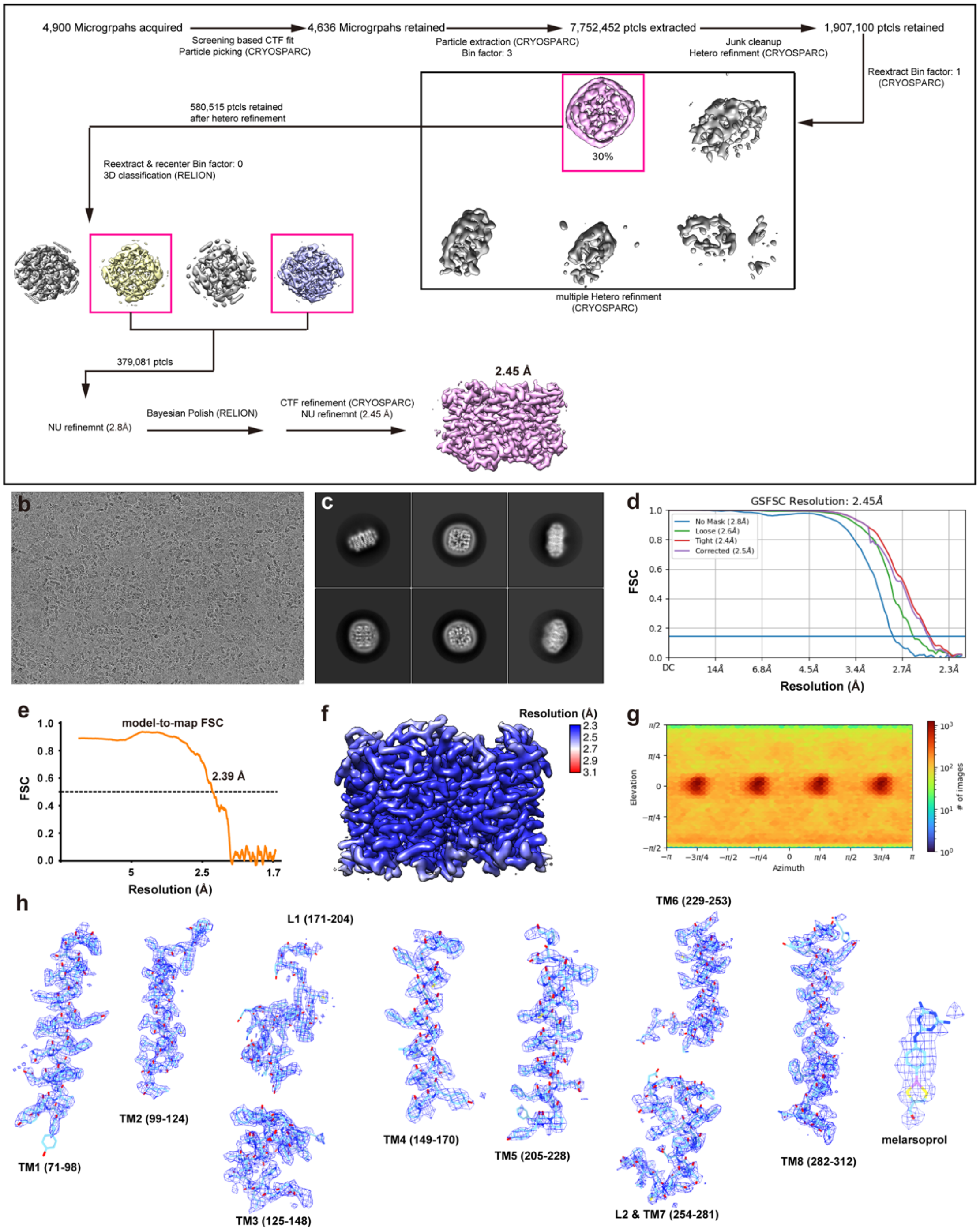


**Supplementary Fig. 4 cryo-EM data processing and reconstruction of TbAQP2-melarsoprol complex.** **a**, Flow charts outlining the cryo-EM processing workflows. **b** and **c**, Representative cryo-EM micrographs and 2D class averages. **d-g**, Half-map FSC curves, map-to-model FSC curves, Estimations of local resolution, and the angular distributions of particles used in the final reconstructions are shown. **h**, Cryo-EM density. Densities (surface rendering, 8 s contour) for indicated regions are shown in the context of the atomic model (sticks).

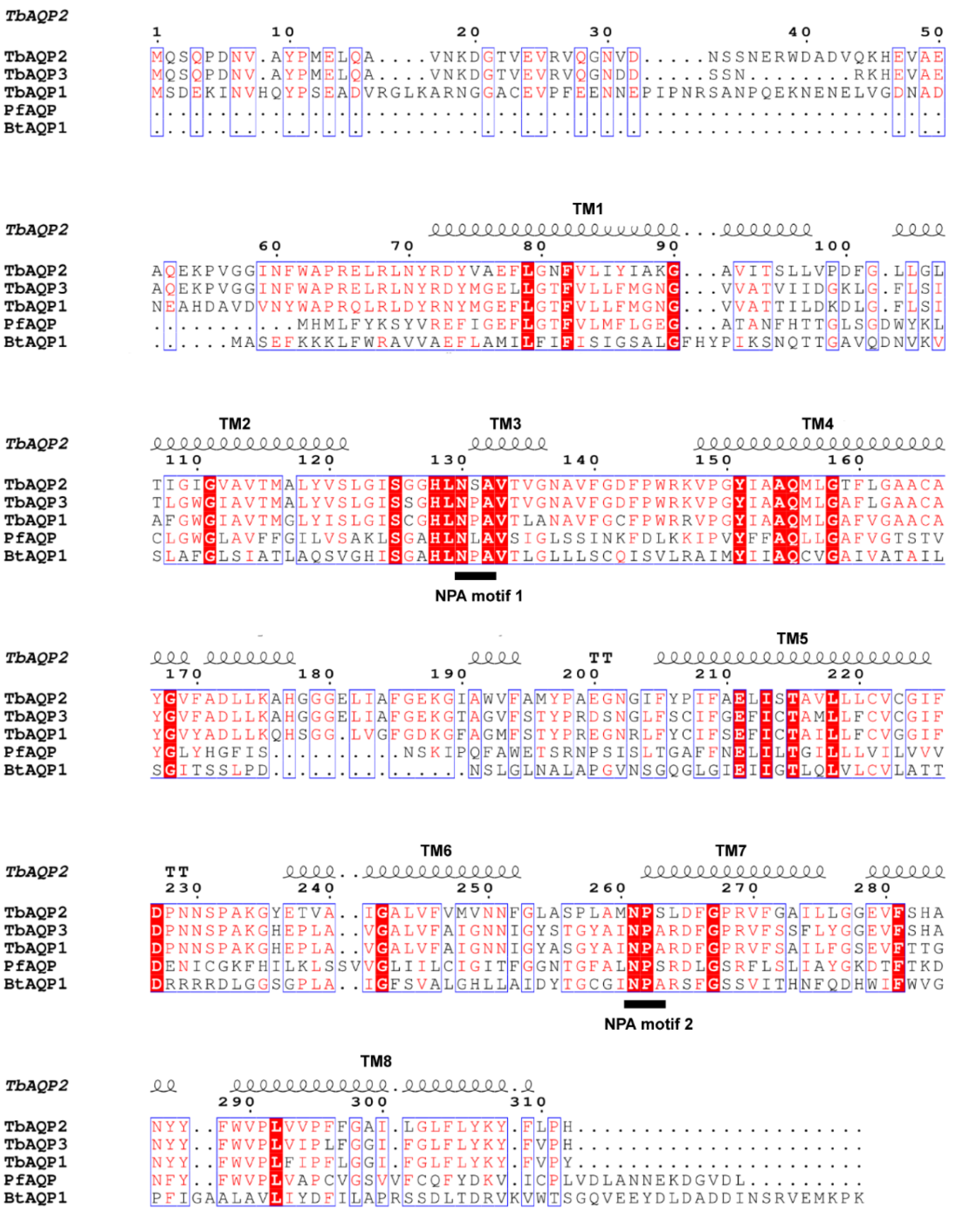

**Supplementary Fig. 5 Sequence comparison of aquaporin proteins.**

The amino acid sequences of *T. brucei* AQP1, *T. brucei* AQP2, *T. brucei* AQP3, *P. falciparum* AQP, and *B. taurus* proteins are aligned and colored according to the ClustalW convention (UniProt accession numbers: Q6ZXT4, Q6ZXT3, Q6ZXT2, Q8WPZ6 and P47865, respectively). TM helixes and NPA motifs are indicated and labeled.

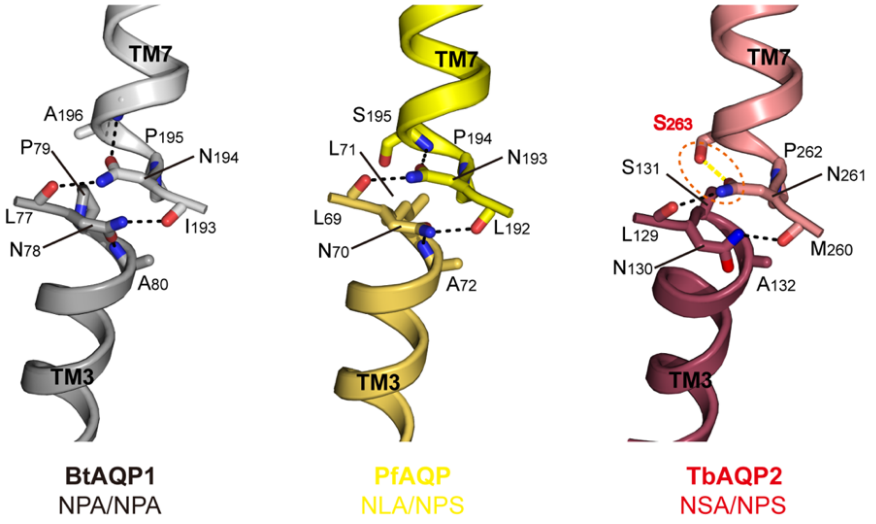


**Supplementary Fig. 6 "fireman's grip-like" structures of the NPA/NPA motifs.**

Cartoon representations of the TM3 & TM7 of BtAQP1 (grey, PDB:1J4N), PfAQP (yellow, PDB:3C02) and TbAQP2 (apo, pink). NPA/NPA motifs and residues stabilizing the "fireman's grip-like" structures are drawn as sticks. The dashed oval highlights the hydrogen bond between $N_{261}$ and $S_{263}$ of TbAQP2 which has not been observed in other AQPs.

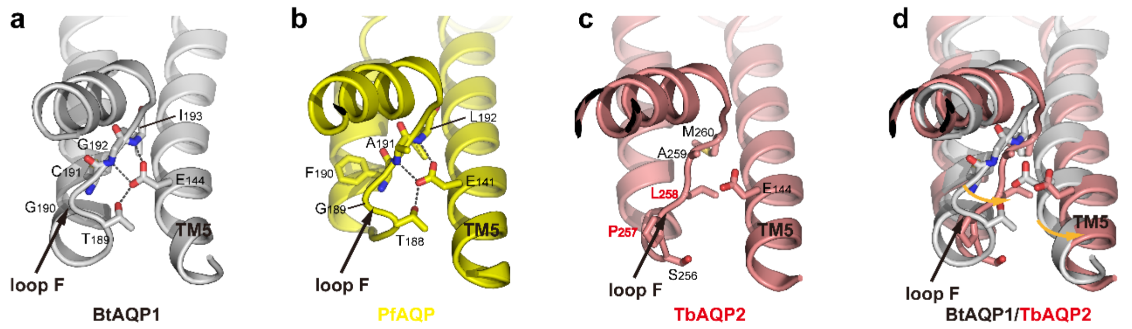


**Supplementary Fig. 7 The arrangement of loop F and TM5.**

**a, b and c,** Cartoon representations of BtAQP1 (grey, PDB:1J4N), PfAQP (yellow, PDB:3C02) and TbAQP2 (apo, pink), respectively. The residues stabilizing the interactions of loop F and TM5 are drawn as sticks. **d**, Superposition of TbAQP2 (pink) with BtAQP1 (grey) reveals the outward rearrangement of loop F and TM5 of TbAQP2.

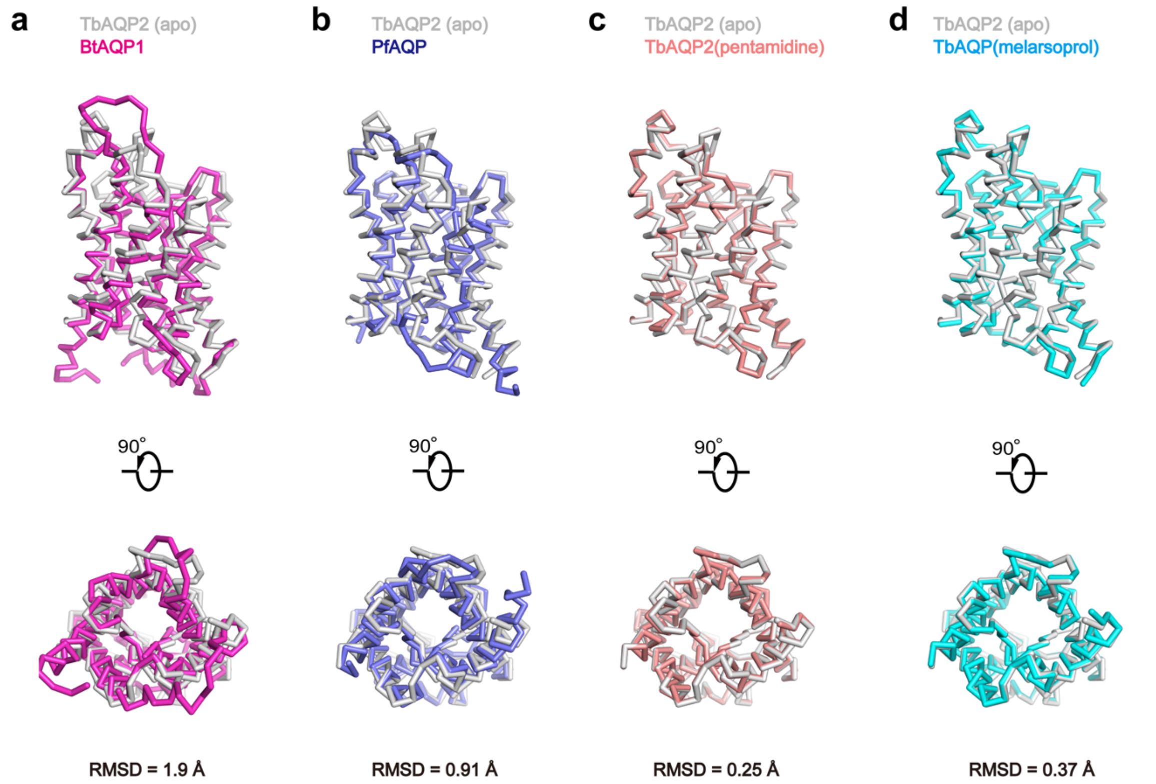


**Supplementary Fig. 8 Comparisons of TbAQP2, BtAQP1 and PfAQP in apo and substrate-bound conformations.**

**a** and **b**, Superposition of TbAQP2 (apo; grey) with BtAQP1 (magentas, PDB:1J4N) and PfAQP (blue, PDB:3C02). The RMSD is 1.9 Å and 0.91 Å (for Cα atoms), respectively. Two orthogonal views are shown. **c** and **d**, Superposition of TbAQP2 in apo conformation (grey) with pentamidine-bound (pink), and melarsoprol-bound (cyan) conformations. The RMSD is 0.25 Å and 0.37 Å (for Cα atoms), respectively. Two orthogonal views are shown.

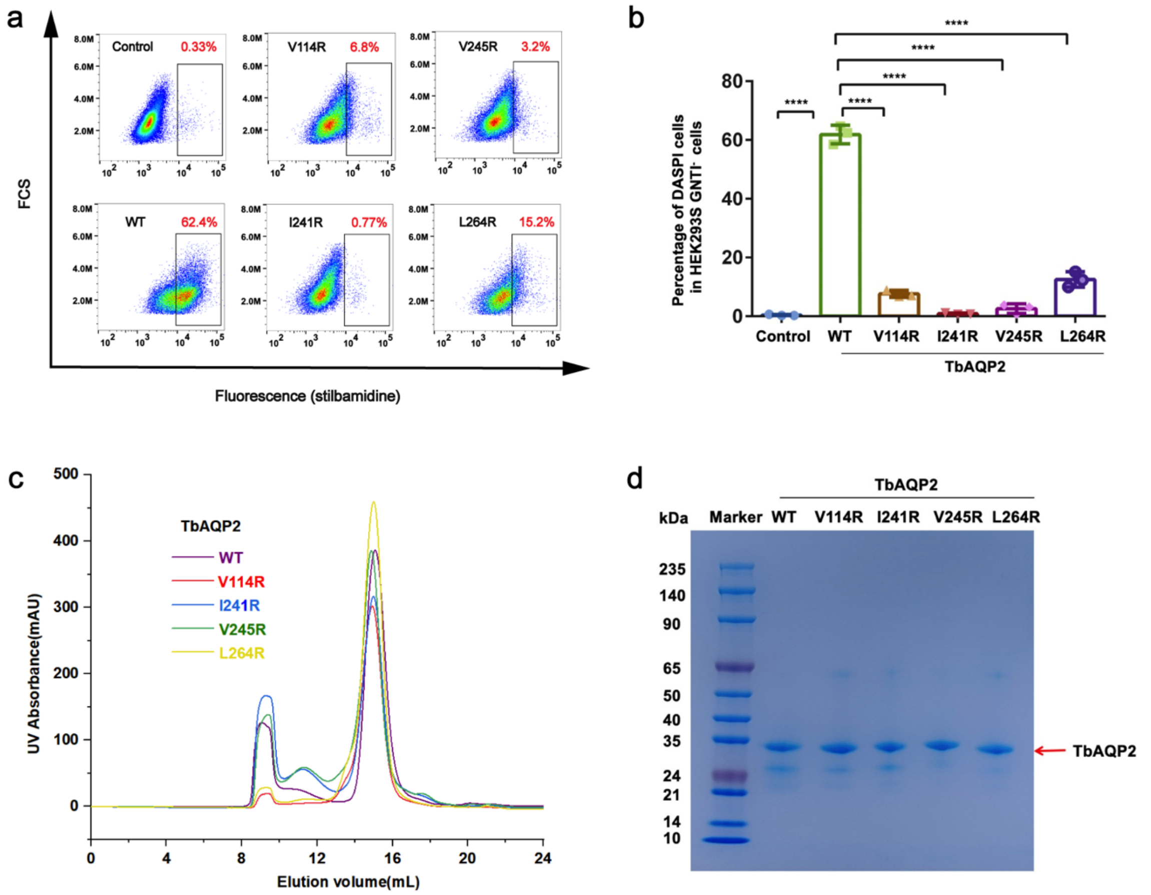


**Supplementary Fig. 9 Flow cytometry analysis of HEK293 cells expressing wild-type TbAQP2 and mutants.**

**a**, the percentage of stilbamidine in HEK293S $GNTI^{-}$ cells was analyzed by flow cytometry. A representative chart from a total of three experiments is presented, and fluorescence was assessed at an excitation wavelength of 405 nm and an emission wavelength of 445±45 nm. **b**, Statistic analysis of the stilbamidine-positive percentages of uninfected cells, $TbAQP2^{WT}$, $TbAQP2^{V114R}$, $TbAQP2^{I241R}$, $TbAQP2^{V245R}$ and $TbAQP2^{L264R}$ infected cells, respectively. All the data were presented as the mean ± SD (standard deviation) from 3 independent experiments. ***, $p < 0.0001$ compared with controls or mutant using Student's t-test. **c**, Size-exclusion chromatography results of wild-type and various mutant TbAQP2, i.e., V114R, I241R, V245R, and V264R. **d**, Representative of purified wild-type and various mutant TbAQP2 detected by SDS-PAGE.

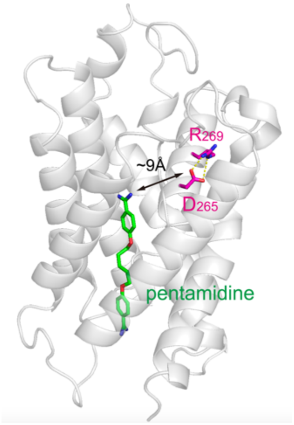


**Supplementary Fig. 10 D265 forms a salt bridge with a neighboring R269.**

TbAQP2 is shown as ribbons; pentamidine, D265 and R269 are drawn as sticks. The distance between the carboxyl group of D265 to the amide group of pentamidine is ~9 Å.

**Supplementary Movie: Molecular dynamics simulation of pentamidine permeation through TbAQP2 under an applied voltage (a 100 ns fragment of the time trace)**.

| | Substrate free<br>PDB: 8JY7<br>EMD-36722 | Pentamidine-bound<br>PDB: 8JY8<br>EMD-36723 | Melarsoprol-bound<br>PDB: 8JY6<br>EMD-36721 |
|---|---|---|---|
| **Data collection and processing** | | | |
| Microscope | FEI Titan Krios | FEI Titan Krios | FEI Titan Krios |
| Camera | Gatan K3 | Gatan K3 | Gatan K3 |
| Magnification | 22,500× | 22,500× | 22,500× |
| Voltage (kV) | 300 | 300 | 300 |
| Electron exposure ($e^{-}/Å^2$) | 71 | 71 | 71 |
| Defocus range (µm) | −1.0 ~ −2.3 | −1.0 ~ −2.3 | −1.0 ~ −2.6 |
| Pixel size (Å) | 1.064 (0.532)* | 1.064 (0.532)* | 1.064 (0.532)* |
| Software | RELION 3.1, cryoSPARC v2 | RELION 3.1, cryoSPARC v2 | RELION 3.0, cryoSPARC v2 |
| Symmetry imposed | C4 | C4 | C4 |
| Initial particle images (no.) | 325,497 | 1,883,669 | 1,907,100 |
| Final particle images (no.) | 153,589 | 539,330 | 379,081 |
| Rsolution (FSC = 0.143, Å) | 3.0 | 2.45 | 2.45 |
| Density modified CC (0.5, Å) | 2.90 | 2.40 | 2.41 |
| Local map resolution range (Å) | 2.5-4.5 | 2.0-3.5 | 2.0-3.5 |
| **Refinement** | | | |
| Software | Phenix 1.13 real-space-refine | Phenix 1.13 real-space-refine | Phenix 1.13 real-space-refine |
| Initial model (PDB code) | N/A | N/A | N/A |
| Model resolution (FSC=0.5/0.143, Å) | 2.94/2.39 | 2.40/2.14 | 2.39/2.14 |
| FSC threshold 0.5 | | | |
| Map sharpening *B* factor ($Å^2$) | -30 | -30 | -30 |
| Model composition | | | |
| Non-hydrogen atoms | 7,292 | 7,416 | 7,404 |
| Protein residues ($^{\#}$) | 972 (69-311) | 972 (69-311) | 972 (69-311) |
| Ligands | | 4(PNT) | 4 (Mel) |
| Water | 0 | 0 | 0 |
| *B* factors ($Å^2$) | | | |
| Protein | 45.48 | 34.39 | 32.18 |
| Ligand | | 38.74 | 42.75 |
| R.m.s. deviations | | | |
| Bond lengths (Å) | 0.004 | 0.004 | 0.002 |

| | | | |
|---|---|---|---|
| Bond angles (°) | 0.720 | 0.877 | 0.476 |
| Validation | | | |
| MolProbity score | 2.38 | 1.30 | 1.83 |
| Clashscore | 11.99 | 2.09 | 10.98 |
| Poor rotamers (%) | 3.30 | 1.64 | 2.19 |
| Ramachandran plot | | | |
| Favored (%) | 94.17 | 97.10 | 97.93 |
| Allowed (%) | 5.83 | 2.90 | 2.07 |
| Disallowed (%) | 0.00 | 0.00 | 0.00 |

**Supplementary Table 1** Data collection, refinement, and validation statistics. *Super-resolution pixel size; #Starting and ending residues.
