# Supplementary material for: Structural insights into drug transport by an aquaglyceroporin
Source: Nat Commun. 2024 May 11;15:3985. doi: 10.1038/s41467-024-48445-4 (PMC11088622; doi:10.1038/s41467-024-48445-4)
Supplement: Supplementary file 3 — Description of Additional Supplementary Files [file 41467_2024_48445_MOESM3_ESM.pdf]

Description of Additional Supplementary Files

File Name: Supplementary Movie

Description: Molecular dynamics simulation of pentamidine permeation through TbAQP2 under an applied voltage (a 100 ns fragment of the time trace)
